# Succination of Protein Thiols in Human Brain Aging

**DOI:** 10.3389/fnagi.2020.00052

**Published:** 2020-03-06

**Authors:** Mariona Jové, Irene Pradas, Natalia Mota-Martorell, Rosanna Cabré, Victoria Ayala, Isidre Ferrer, Reinald Pamplona

**Affiliations:** ^1^Department of Experimental Medicine, University of Lleida-Lleida Biomedical Research Institute (UdL-IRBLleida), Lleida, Spain; ^2^Institute of Neuropathology, Bellvitge University Hospital, University of Barcelona, Biomedical Research Institute of Bellvitge (IDIBELL), L’Hospitalet de Llobregat (Barcelona), Barcelona, Spain; ^3^Center for Biomedical Research on Neurodegenerative Diseases (CIBERNED), ISCIII, Madrid, Spain

**Keywords:** cysteine, fumarate, loss-of-function, mitochondrial stress, posttranslational modification (PTM), 2-(S-succino)cysteine (2SC), thiol groups

## Abstract

Human brain evolution toward complexity has been achieved with increasing energy supply as the main adaptation in brain metabolism. Energy metabolism, like other biochemical reactions in aerobic cells, is under enzymatic control and strictly regulated. Nevertheless, physiologically uncontrolled and deleterious reactions take place. It has been proposed that these reactions constitute the basic molecular mechanisms that underlie the maintenance or loss-of-function of neurons and, by extension, cerebral functions during brain aging. In this review article, we focus attention on the role of the nonenzymatic and irreversible adduction of fumarate to the protein thiols, which leads to the formation of S-(2-succino)cysteine (2SC; protein succination) in the human brain. In particular, we first offer a brief approach to the succination reaction, features related to the specificity of protein succination, methods for their detection and quantification, the bases for considering 2SC as a biomarker of mitochondrial stress, the succinated proteome, the cross-regional differences in 2SC content, and changes during brain aging, as well as the potential regulatory significance of fumarate and 2SC. We propose that 2SC defines cross-regional differences of metabolic mitochondrial stress in the human brain and that mitochondrial stress is sustained throughout the healthy adult lifespan in order to preserve neuronal function and survival.

## Introduction

A posttranslational modification (PTM) is a chemical-biological modification that takes place to one or more amino acids on a protein after that protein has been translated by a ribosome (Walsh, 2006). The PTM of proteins is a mechanism in all living organisms and cell types which helps to regulate their physiological processes. PTMs allow cells to expand their protein function and coordinate their signaling networks. These modifications have an enzymatic origin and can be either reversible or irreversible, and they dynamically alter the structure, stability, and functions of proteins in the cells (Walsh, 2006). Examples of PTMs include, glycosylation, phosphorylation, acetylation, methylation, ubiquitylation, and sumoylation, among several others. At present, around 400 PTM types have been described as affecting a significant fraction of cell proteome (Lothrop et al., 2013). PTMs play a structural role in determining protein folding, interaction with ligands and other proteins, and targeting specific subcellular compartments, as well as playing a functional role in regulating protein turnover, catalytic activity, and signaling function, among others (Walsh, 2006; Prabakaran et al., 2012; Santos and Lindner, 2017).

In contrast to enzymatic PTMs, non-enzymatic PTMs include chemical modifications mediated by reactive compounds on proteins, which is a central event in endogenous cell chemical damage. However, the role of several types of these non-enzymatic and mostly irreversible modifications remains to be elucidated. Examples of non-enzymatic PTMs are glycation, glyco- and lipoxidation, nitrosylation, and oxidation/reduction (Thorpe and Baynes, 2003; Golubev et al., 2017). The sulfhydryl group of cysteine (Cys) residues in proteins are also the target of several PTM types, including adduction to electrophiles, glutathionylation, nitrosylation, sulphonation, sulphenation, and sulphydration (Couvertier et al., 2014). In this review article, we will focus on the adduction of fumarate to Cys residues in proteins, designated as succination, its occurrence in the brain, and its putative role in aging.

## The Succination Reaction

**Figure 1:**
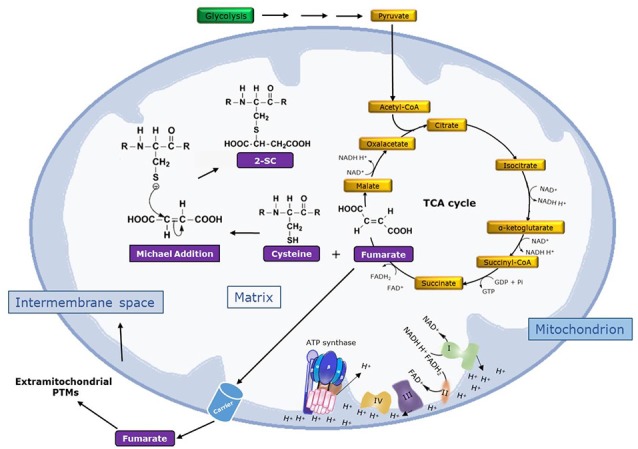
Mechanism of formation of 2SC. The figure shows the conversion of glucose to pyruvate in the glycolysis pathway. Then, pyruvate is converted to acetyl-CoA, which enters the tricarboxylic acid (TCA) cycle, and the reducing equivalents (NADH and FADH2) generated from glycolysis and the TCA cycle enter the mitochondrial electron transport chain (ETC). So, glycolysis, the TCA cycle, and the ETC are integrated pathways to catabolize energy substrates and drive ATP synthesis *via* the complex V (ATP synthase). Complexes I–IV of the ETC and ATP synthase are shown. Importantly, the nucleophilic addition of the TCA cycle metabolite fumarate to cysteine yields 2SC by a Michael addition reaction (R indicates peptide chain) in a non-enzymatic reaction called protein succination. Fumarate can also be exported outside mitochondria by carriers, potentially inducing 2SC formation in other cell proteins and even at the extracellular level.

The adduction of fumarate to the sulfhydryl group in the side chain of Cys residues in proteins *via* a Michael-like reaction at physiological pH leads to the formation of S-(2-succino)cysteine (2SC; Alderson et al., 2006). This non-enzymatic and irreversible reaction, which generates a thioether bond, is called *succination* of proteins in order to distinguish it from *succinylation*, which is a reversible enzymatic reaction that leads to the formation of amide, ester, or thioester bonds (Alderson et al., 2006; Merkley et al., 2014). Chemically, succination preferentially targets cysteine residues with low pKa values (as low as 3–4), such as those located in active sites of proteins, in contrast to the pKa of about nine present in the free thiol group of cysteine and glutathione (Merkley et al., 2014). Succination is specific for cysteine and no modification was detected in *in vitro* incubations of fumarate with other candidate amino acids like lysine and histidine in protein models, nor is it modified like N-acetylcysteine or N-acetylhistidine (Merkley et al., 2014). Figure 1 shows a scheme for the formation of 2SC by the reaction of fumarate with cysteine.

The cysteine-fumarate adduct 2SC was the first described chemical modification of proteins by a metabolite intermediate in the Krebs cycle (Alderson et al., 2006). These findings identify fumarate as an endogenous electrophile and suggest a potential role for fumarate in the regulation of cell metabolism. In fact, it has been proposed that 2SC is a biomarker of mitochondrial and oxidative stress (Blatnik et al., 2008a).

## Structural and Functional Features of the Nonenzymatic Modification of Proteins by Succination

The analysis of the succinated proteome with 2SC sites applying theoretical approaches to analyze sequence- and 3D-structure-based features is an excellent source of information in order to learn about the molecular mechanism, features related to specificity, and the role of protein succination. In this context, a recent study by Miglio et al. (2016) collected and examined a total of 182 succinated proteins from three reports (Ternette et al., 2013; Merkley et al., 2014; Yang et al., 2014) including additional information on 205 modified and 1,750 non-modified sites. Notably, the set of modified proteins showed a wide distribution and, although proteins are predominantly located into the mitochondria, damaged proteins from other locations like cytosol, endoplasmic reticulum, and the nucleus were also detected. These observations demonstrate that fumarate can reach other cellular and probably extracellular compartments, likely due to the fact that fumarate can be exported from mitochondria through specific carriers (Figure 1). Reinforcing this, a succinate/fumarate mitochondrial transporter (SFC1) present only in *S. cerevisiae* and a mitochondrial dicarboxylate carrier (DIC) present in animal tissues (including brain) have been described, highlighting the relevance of the extramitochondrial transport of fumarate and the importance of deciphering its exact molecular mechanism (Fiermonte et al., 1999; Lin et al., 2011). Thus, fumarate seems to behave like a long-lived reactive compound and can be more damaging because it has a more prominent global impact in altering a wider collection of protein targets beyond mitochondria due to their transient nature.

The analysis of the succinated proteome also suggests that protein modification is not restricted to proteins of a specific metabolic-biological process or molecular function, suggesting that succination may have wide-ranging effects on the structural cell components and the regulation of metabolism. Thus, proteins involved in cytoskeleton organization, ion (iron, zinc, and copper) homeostasis, metabolic processes (amino acids, carbohydrates, fatty acids, and nucleotides metabolism), protein synthesis, redox homeostasis, RNA processing, and signaling pathways are modified (Nagai et al., 2007; Ternette et al., 2013; Merkley et al., 2014; Yang et al., 2014). Interestingly, more than 80% of succinated proteome showed a single 2SC site, with the 2SC sites ranging from 1 to 3 per protein, and the overall relative abundance of modifiable sites is 10.8% (Miglio et al., 2016). These findings demonstrate the heterogeneity of the modified proteins, reinforces the non-specific character of this PTM type and confirms that only certain Cys residues can react with fumarate to generate 2SC. Consequently, these observations confirm the existence of features of Cys residues that govern the specificity of 2SC formation and confirm the selectivity of the modification for particular proteins.

Finally, the analysis of 2SC site features demonstrated that modified and non-modified sites may be differentiated when the accessibility of the sulphur atoms and the amino acid composition of the Cys-flaking peptides is analyzed, whereas these were not distinguishable when the “acid dissociation constant value of the sulfhydryl groups,” the “hydrophobicity of the Cys-flaking peptides,” and the “secondary structure of the Cys-containing segments” were compared (Miglio et al., 2016).

## Methods for Detecting Succination of Proteins

Currently, mass spectrometry (MS)-based analysis and antibody-based detection techniques are the main methods used to detect and analyze protein succination. However, the MS approach is the only available tool for global or large-scale analysis.

The availability of specific antibodies that can recognize modified amino acid residues within a peptide or protein conditions antibody-based methods. These antibodies can be monoclonal or polyclonal and are developed against either the modified amino acid or peptide/protein. Later, the quantification of PTMs on peptide/protein using antibody-based techniques can be done with two methods: absorbance/fluorescence-based ELISA and chemiluminescence-based Western blotting. In any case, the detection of PTMs absolutely depends on the recognition site of the antibody applied. For succinated proteins a characterized polyclonal antibody against 2SC for immunoblot analysis, two-dimensional PAGE analysis, and immunohistochemistry has been systematically used (Nagai et al., 2007; Frizzell et al., 2009, 2012; Bardella et al., 2011; Thomas et al., 2012; Manuel and Frizzell, 2013; Ternette et al., 2013; Merkley et al., 2014; Piroli et al., 2014, 2016; Yang et al., 2014).

MS detection of specific 2SC sites is based on mass changes. Using liquid chromatography-electrospray ionization-MS (LC-ESI-MS)-based proteomics analysis, subsequent fragmentation and sequencing of a relevant peptide has been used to identify specific site of succination (Blatnik et al., 2008a; Frizzell et al., 2009; Ternette et al., 2013; Merkley et al., 2014; Piroli et al., 2014, 2016; Yang et al., 2014). Yet, technical challenges hamper MS-based investigation of the succination of proteins, many of them linked to or derived from the low-abundance of this non-enzymatic modification (Merkley et al., 2014).

**Figure 2:**
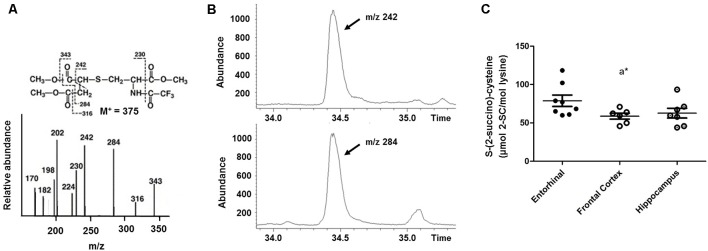
Detection and quantification by GC/mass spectrometry (MS)/MS of 2SC in human brain samples. 2SC can be determined as trifluoroacetic acid methyl ester (TFAME) derivatives in acid hydrolyzed, delipidated, and reduced brain protein samples by GC/MS. **(A)** Structure, mass spectrum, and proposed fragmentation patterns of TFAME derivative of natural 2SC. **(B)** Detection of 2SC in human brain proteins. Selected ion chromatograms for brain proteins showing the m/*z* = 242 and m/*z* = 284 ions. **(C)** 2SC quantification in different regions of the human brain (adapted from Cabré et al., 2016 with permission). *p < 0.05. A significant difference with respect to the entorhinal cortex.

MS-based techniques—specifically gas chromatography-MS (GC/MS)—can also be used to detect and measure the steady-state level of 2SC at the subcellular, cellular, tissue, and organ levels (Nagai et al., 2005, 2007; Alderson et al., 2006; Blatnik et al., 2008a; Frizzell et al., 2009; Cabré, 2015; Gomez et al., 2015; López-González et al., 2015; Cabré et al., 2016, 2017; Martínez-Cisuelo et al., 2016; Piroli et al., 2016; Naudí et al., 2017). In this approach, 2SC can be determined as TFAME (trifluoroacetic acid methyl esters) derivatives in protein samples (around 0.5–1 mg of protein final concentration) previously hydrolyzed in acidic conditions and using a GC coupled to an MS, an HP-5MS column (30 m × 0.25 mm × 0.25 μm), and a specific temperature program ranging from 110°C to 300°C. Later, quantification can be made by internal and external standardization using standard curves created from mixtures of deuterated and non-deuterated standards. Analyses can be carried out with SIM-GC/MS (selected ion-monitoring GC/MS). In this case, the ions used for detection and quantification are lysine and [^2^H_8_]lysine, m/z 180 and 187, respectively; and 2SC and [^2^H_2_]2SC, m/z 284 and 286, respectively. Thus, the amount of product can be expressed as mili- or micromoles of 2SC per mol of lysine. Figure 2 shows the detection and quantification by GC/MS/MS of 2SC in a human brain sample.

## Protein Succination in Physiological and Pathological Models

The detection of the protein adduct 2SC in a biological system was originally demonstrated in human erythrocytes (globin; Nagai et al., 2005), plasma proteins (albumin), and skin collagen (Alderson et al., 2006). Interestingly, 2SC increases in human skin collagen with age (Alderson et al., 2006). Later, the presence of this post-translational Cys modification type was extended to three pathological models: diabetes/obesity, fumarate hydratase (FH)-related diseases, and Leigh syndrome. In all these models, increased steady-state levels of 2SC were found due to increased levels of fumarate ascribed to dysfunctional mitochondrial metabolic activity. More specifically, increased levels of 2SC were found in erythrocytes and adipocytes (murine 3T3-L1 adipocytes) cultured in high glucose medium (30 mM, compared with the physiological concentration of 5 mM; Nagai et al., 2005, 2007; Frizzell et al., 2012; Manuel and Frizzell, 2013; Piroli et al., 2014; Manuel et al., 2017), as well as in tissues (e.g., adipose tissue, skeletal muscle or urine) from streptozotocin-treated rats—a model of type 1 diabetes—(Alderson et al., 2006; Blatnik et al., 2008b; Thomas et al., 2012), db/db (leptin receptor deficient; Frizzell et al., 2009; Thomas et al., 2012; Piroli et al., 2014; Manuel et al., 2017), Akita diabetic mice (Thomas et al., 2012), ob/ob (leptin deficient; Thomas et al., 2012), and diet-induced obese mice (Thomas et al., 2012). In all these models, the proposed underlying molecular mechanism is related to the excess of nutrients and implies an elevated ATP/ADP and NADH/NAD+ ratios as well as an elevated mitochondrial membrane potential. The increased NADH/NAD+ ratio down-regulates oxidative phosphorylation, leading to sustained accumulation of mitochondrial metabolites including fumarate, which, in turn, gives rise to protein succination (Frizzell et al., 2012).

An increased concentration of succinated proteins has also been reported in a pathological model based on FH enzyme loss-of-function (Bardella et al., 2011; Ternette et al., 2013; Yang et al., 2014). FH catalyzes the reversible conversion of fumarate to malate in the tricarboxylic acid cycle (TCA or Krebs cycle). In this model, protein succination is involved in FH-related carcinogenesis (Bardella et al., 2011; Ternette et al., 2013; Yang et al., 2014), and more importantly, has been proposed as acting as an oncometabolite (Yang et al., 2012).

Finally, increased levels of 2SC have also been described in the brainstem of *Ndufs*4 knockout mice (a model of Leigh syndrome; Piroli et al., 2016), suggesting a potential role for protein succination in the physiopathogenesis of this mitochondrial disorder. Ndufs4 is a subunit of the mitochondrial complex I which plays a key structural role, allowing the correct assembly, stability, and activity of this complex and, consequently, the correct function of the mitochondrial electron transport chain (ETC) and the subsequent energy production. Mutations in *Ndfus*4 produce a defective mitochondrial energy metabolism, and animals develop fatal encephalomyopathy with premature death which recapitulates the signs of Leigh syndrome.

Together, these observations indicate that increased 2SC content in tissue proteins is a direct consequence of increased intracellular fumarate concentration. For this reason, 2SC has been proposed as a biomarker of metabolic stress, and more specifically, of mitochondrial stress (Nagai et al., 2007; Blatnik et al., 2008a; Frizzell et al., 2009, 2012; Thomas et al., 2012).

Succination has been reported in almost all tissues analyzed using immunoblotting and mass spectrometric techniques, although the degree of modification varies significantly among them. Thus, the detection of 2SC by immunoblotting shows that there are tissue-specific differences in the renal medulla and cortex, testes, lungs, soleus, gastrocnemius, heart, liver, spleen, and fat (suprarenal, subcutaneous, and epididymal adipose tissue; Thomas et al., 2012). In line with this, the presence of 2SC was also described in liver mitochondria from rats and mice (Gomez et al., 2015; Martínez-Cisuelo et al., 2016), and in several brain regions from humans, rat, and mice (Cabré, 2015; López-González et al., 2015; Cabré et al., 2016; Naudí et al., 2017) using MS techniques. Table 1 shows the steady-state levels of 2SC measured by GC/MS in different brain regions, tissues, and species. Notably, the steady-state level of 2SC is in the range of micromoles 2SC/mol lysine, clearly indicating the low abundance of this post-translational modification at the tissue level.

**Table 1:** Steady-state levels of 2SC measured by gas chromatography/mass spectrometry (GC/MS) in different brain regions, tissues and species.

Tissue	Species	Concentration	Reference
Spinal cord	Human	0.101	Naudí et al. (2017)
Medulla oblongata	Human	0.112	Naudí et al. (2017)
Cerebellum	Human	0.060	Naudí et al. (2017)
Substantia nigra	Human	0.115	Naudí et al. (2017)
Thalamus	Human	0.084	Naudí et al. (2017)
Amygdala	Human	0.086	Naudí et al. (2017)
Striatum	Human	0.080	Naudí et al. (2017)
Entorhinal cortex	Human	0.075	Naudí et al. (2017)
Entorhinal cortex	Human	0.078	Cabré et al. (2016)
Hippocampus	Human	0.062	Naudí et al. (2017)
Hippocampus	Human	0.063	Cabré et al. (2016)
Temporal cortex	Human	0.044	Naudí et al. (2017)
Occipital cortex	Human	0.046	Naudí et al. (2017)
Frontal cortex	Human	0.058	Naudí et al. (2017)
Frontal cortex	Human	0.059	Cabré et al. (2016)
Spinal cord (lumbar)	Rat	0.019	Cabré (2015)
Spinal cord (thoracic)	Rat	0.015	Cabré (2015)
Spinal cord (cervical)	Rat	0.018	Cabré (2015)
Medulla oblongata	Rat	0.040	Cabré (2015)
Pons	Rat	0.048	Cabré (2015)
Cerebellum	Rat	0.039	Cabré (2015)
Olfactory bulb	Rat	0.051	Cabré (2015)
Thalamus	Rat	0.042	Cabré (2015)
Hypothalamus	Rat	0.047	Cabré (2015)
Striatum	Rat	0.060	Cabré (2015)
Hippocampus	Rat	0.047	Cabré (2015)
Temporal cortex	Rat	0.041	Cabré (2015)
Occipital cortex	Rat	0.044	Cabré (2015)
Frontal cortex	Rat	0.028	Cabré (2015)
Somatosensory cortex	Mouse	0.062	López-González et al. (2015)
Hippocampus	Mouse	0.079	López-González et al. (2015)
Spinal cord (lumbar)	Mouse	0.042	López-González et al. (2015)
Erythrocytes	Human	0.016	Nagai et al. (2005)
Plasma proteins (albumin)	Human	0.6	Alderson et al. (2006)
Skin collagen	Human	0.06	Alderson et al. (2006)
Liver mitochondria	Mouse	0.088	Martínez-Cisuelo et al. (2016)
Epididymal adipose tissue	Mouse	0.060	Frizzell et al. (2009)
Liver mitochondria	Rat	0.083	Gomez et al. (2015)
Skeletal muscle	Rat	0.007	Alderson et al. (2006)
Skeletal muscle	Rat	0.007	Blatnik et al. (2008a)
Urine	Rat	120*	Alderson et al. (2006)

Data are from healthy adult individuals or specimens. Units: mmol 2SC/mol lysine. *nmol/day.

## S-(2-Succino)Cysteine (2SC) in Human Brain Tissue

**Figure 3:**
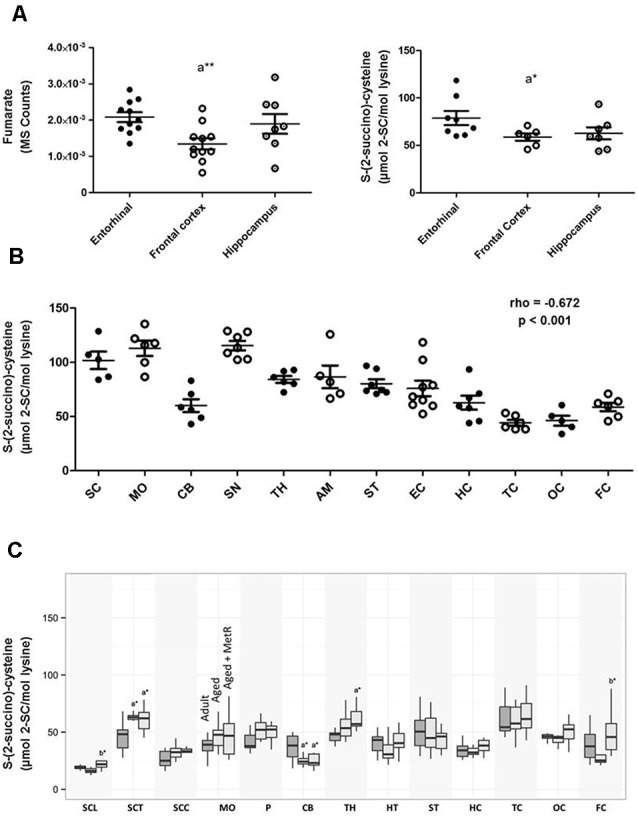
**(A)** Concentrations of fumarate (left) and 2SC (right) in different regions of the adult human cerebral cortex. Fumarate was detected and quantified with TQMS. The steady-state level of 2SC was measured with GC-MS. *p < 0.05; **p < 0.01. A significant difference with respect to the entorhinal cortex (adapted from Cabré et al., 2016 with permission). **(B)** Steady-state levels of 2SC in 12 regions of the adult healthy human central nervous system show a significant inverse correlation following a caudal-cranial axis (*σ*_(rho)_ = −0.672, *p* < 0.001; adapted from Naudí et al., 2017 with permission). Values are mean ± SEM from 5–8 samples for each region. Abbreviations: SC, spinal cord; MO, medulla oblongata; CB, cerebellum; SN, substantia nigra; TH, thalamus; AM, amygdala; ST, striatum; EC, entorhinal cortex; HC, hippocampus; TC, temporal cortex; OC, occipital cortex; FC, frontal cortex. rho (σ), Spearman’s rank correlation coefficient. **(C)** Effects of aging and methionine restriction in old age on the concentration of 2SC in 13 different brain regions of rat. Values are mean ± SEM from 10 animals per group for each region. Abbreviations: SC, spinal cord (L, lumbar; T, thoracic; C, cervical); MO, medulla oblongata; P, pons; CB, cerebellum; TH, thalamus; HT, hypothalamus; ST, striatum; HC, hippocampus; TC, temporal cortex; OC, occipital cortex; FC, frontal cortex.

Human brain evolution is closely associated with the expansion of its size and complexity, a key condition for the appearance of cognitive functions, demanding adaptations in brain energy metabolism (Somel et al., 2013; Cabré et al., 2016). The main energy provider for neurons and glial cells is the mitochondrion. Consequently, it is reasonable to propose that these cell types can be particularly susceptible to succination of proteins. Effectively, the available evidence demonstrates that protein succination occurs in brain tissue as in other cell systems. Thus, the formation of 2SC has been described in several brain regions in mouse (López-González et al., 2015; Piroli et al., 2016), rat (Cabré, 2015) and human (Cabré et al., 2016, 2017; Naudí et al., 2017). By using immunoblotting, 2SC was detected in several mouse brain areas including olfactory bulb, cortex, striatum, cerebellum, and brainstem (Piroli et al., 2016). By using MS, 2SC was analyzed in somatosensory cortex, hippocampus, and spinal cord (lumbar) in mouse (López-González et al., 2015); cortex (temporal, occipital, and frontal), hippocampus, striatum, hypothalamus, thalamus, olfactory bulb, cerebellum, pons, medulla oblongata, and spinal cord (lumbar, thoracic and cervical) in rat (Cabré, 2015); and cortex (occipital, temporal, and frontal), hippocampus, entorhinal cortex, striatum, amygdala, substantia nigra, thalamus, cerebellum, medulla oblongata, and spinal cord in human (Cabré et al., 2016; Naudí et al., 2017). Table 1 shows the steady-state levels of 2SC measured by GC/MS in different brain regions and species. So, 2SC concentration ranged from 0.042 to 0.079 mmoles of 2Sc/mol lys in mice, from 0.015 to 0.060 mmoles of 2SC/mol lys in rats (see also Figure 3C), and from 0.044 to 0.112 mmoles of 2SC/mol lys in humans. Notably, the higher 2SC concentration seems to be present in the human brain, likely as an expression of the higher energy demands. This range of protein succination is consistent with the notion of the low abundance of this non-enzymatic PTM. Furthermore, the findings from the immunoblotting approach also confirm the selectivity of the succinated proteins and the presence of cross-regional differences (Piroli et al., 2016), as well the relationship between fumarate content and the degree of protein succination (Cabré et al., 2016; see Figure 3A).

The findings in the human brain are particularly interesting. Thus, the comparison of 12 brain regions (frontal cortex, occipital cortex, temporal cortex, hippocampus, entorhinal cortex, striatum, amygdala, thalamus, substantia nigra, cerebellum, medulla oblongata, and spinal cord) uncovered the existence of cross-regional differences in the steady-state levels of 2SC in the human central nervous system, and, in consequence, it established a region-dependent vulnerability (Naudí et al., 2017; see also Table 1, Figure 3B). Notably, the relationship between 2SC and its distribution following the main subdivisions of the embryonic vertebrate brain according to the cranial-caudal axis (from cerebral cortex to spinal cord) verified that the higher (more cranial) the human brain region, the lower the 2SC content (*σ*_(rho)_ = −0.672, *p* < 0.001; Figure 3B), suggesting a lower mitochondrial stress in the superior human brain regions (Naudí et al., 2017).

## Protein Succination During Human Brain Aging

Brain aging is an endogenous, and deleterious, physiological process characterized by structural and functional changes at different levels of the biological organization, leading to loss of function at the cognitive, regulatory, motor, and sensitive levels (Bishop et al., 2010). Indeed, functional decline and specifically, cognitive loss, are a health challenge for the current century. This challenge is derived from the increased longevity of the general population which attests to the increase in the prevalence of cognitive decline, dementia, and Alzheimer’s disease. In fact, aging is the main cause and risk factor for cognitive deterioration and Alzheimer’s disease in the elderly.

Functional studies have identified phenotypes and signaling pathways that affect and modulate aging in model organisms and brain aging in mammalian species (human included). Among these, are changes in both mitochondrial and synaptic function (Bishop et al., 2010). Importantly, and in contrast to Alzheimer’s disease pathology and other neurodegenerative diseases, human neuronal cells are functional throughout the entire healthy adult lifespan. Consequently, it may be postulated that the maintenance of neuronal function throughout the healthy adult lifespan must be sustained, at least in part, by the ability of neurons to maintain the metabolic conditions at the mitochondrial level under strict control. In this context, 2SC, as a biomarker of mitochondrial stress, can be a good indicator of the mitochondrial metabolic status and aging.

With this premise, different non-enzymatic PTMs were analyzed in the frontal cortex of healthy humans covering an age range from 40 to 90 years old, using MS (Cabré et al., 2017). The results demonstrated increased protein oxidative (aminoadipic- and glutamic- semialdehyde markers) and lipo- and glycoxidative (carboxyethyl- and carboxymethyl-lysine markers) damage in human frontal cortex during the healthy adult lifespan, with a breakpoint at 60 years old (Cabré et al., 2017). Remarkably, this accumulation of oxidative damage with age is selective because when compared with the non-enzymatic modification of cysteine residues (2SC and carboxymethyl-cysteine), no changes were observed. These findings suggest better protection of cysteine residues and their properties by specific and reversible mechanisms such as S-sulfhydration and polysulfidation to prevent cysteine irreversible modification (Takata et al., 2019; Zivanovic et al., 2019), and suggest that in human frontal cortex mitochondrial metabolic stress is strictly sustained within physiological limits throughout the healthy adult lifespan, being a key mechanism in supporting neuronal function and survival (Cabré et al., 2017).

Reinforcing this observation and idea, the measurement of 2SC in different brain regions of adults and aged rats offers very similar results (Cabré, 2015). Specifically, in order to analyze the potential changes induced by age during adult lifespan, 2SC was measured in 14 different brain regions [spinal cord (lumbar, thoracic and cervical), medulla oblongata, pons, cerebellum, olfactory bulb, thalamus, hypothalamus, striatum, hippocampus, and cortex (temporal, occipital, and frontal)] of rats arranged into two age groups: adults (8 months) and aged (26 months). The results demonstrated non-significant changes with aging for all analyzed regions, clearly suggesting that mitochondrial stress is also maintained throughout the adult healthy lifespan (Figure 3C). Interestingly, the application of an anti-aging nutritional intervention like methionine restriction (Pamplona and Barja, 2006) in old age allowed the maintaining of steady-state concentrations of 2SC at adult levels in all brain regions (Cabré, 2015; Figure 3C).

## Potential Regulatory Significance of Fumarate and 2SC

By reacting with nucleophilic sites to proteins involving Cys residues, the Krebs cycle metabolite fumarate generates 2SC. 2SC has been detected, characterized, and localized in the human brain.

The molecular effects of 2SC generation on proteins mostly comprise deleterious structural and functional changes. Thus, the functional consequences of protein succination include a loss of function of the target protein (Blatnik et al., 2008a; Frizzell et al., 2009; Ternette et al., 2013; Piroli et al., 2014). In a similar way to other non-enzymatic PTMs, protein succination can also induce alterations in their physical-chemical properties such as solubility, hydrophobicity, conformation, and charge, among others.

**Figure 4:**
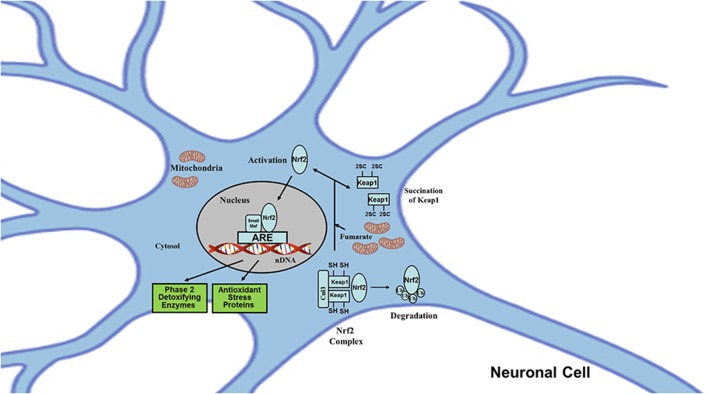
Protein succination may mediate neuroprotection by the activation of signaling pathways modulated by Nrf2. The transcription factor Nrf2 regulates the expression of genes encoding proteins with broad cytoprotective activities and mitochondrial functions. Nrf2 itself is regulated at the level of protein stability. Under baseline physiological conditions, Nrf2 is a short-lived protein that is exposed to incessant turnover *via* ubiquitination and proteasomal degradation. There are three known ubiquitin ligase systems that participate in the removal of Nrf2: (a) Keap1, a substrate adaptor protein for Cullin 3 (Cul3)/Rbx1 ubiquitin ligase; (b) glycogen synthase kinase (GSK)3/β-TrCP-dependent Cul1-based ubiquitin ligase; and (c) E3 ubiquitin ligase Hrd1. In addition to the ubiquitin ligase substrate adaptor protein activity, Keap1 is also the sensor for a wide array of small-molecule activators of Nrf2 (called inducers). Oxidants like reactive oxygen and nitrogen species, electrophiles like carbonyl compounds derived from lipid peroxidation or carbohydrates oxidation, and fumarate are all considered inducers. Inducers block the cycle of Keap1-mediated degradation of Nrf2 by chemically modifying (e.g., by protein succination) specific cysteine residues within Keap1 (Keap1 is a cysteine-rich protein; e.g., rat and mouse have 25 and human 27) or by directly disrupting the Keap1: Nrf2 binding interface. Consequently, Nrf2 is not degraded; it accumulates and translocates to the nucleus, forms a heterodimer with a small Maf protein, binds to antioxidant-response elements (AREs), and initiates transcription.

In contrast, 2SC formation can also mean protein activation when the target protein is structurally and functionally designed to sense and respond to cellular fumarate production. Besides their cytotoxic roles, fumarate and 2SC also exert neuroprotective effects through the activation of adaptive responses specifically designed to decrease the impact of protein damage and relieve mitochondrial stress. One of the mechanisms involved is the antioxidant response signaling pathway Nrf2 (Nuclear factor erythroid 2-related factor 2) subsequent to the succination of Keap1 (Kelch-like ECH-associated protein 1; Figure 4).

Nrf2 is a transcriptional factor that acts as master regulator of the cell antioxidant defenses and the mitochondrial function in order to maintain and adapt redox homeostasis of neuronal and glial cells (Dinkova-Kostova and Abramov, 2015; Johnson and Johnson, 2015; Yamazaki et al., 2015; Baxter and Hardingham, 2016; Vasconcelos et al., 2019). Nrf2 modulates the expression of more than 200 genes by binding to a promoter element called ARE (antioxidant response element) present in these genes. These genes encode for proteins involved in cytoprotection (including antioxidant, anti-inflammatory, detoxification, repair, and removal activities), maintenance of redox homeostasis (biosynthesis of glutathione, thioredoxin, and NADPH), control of mitochondrial ROS (reactive oxygen species) production (by uncoupling proteins), mitochondrial function (ATP synthesis, availability of substrates for ETC, fatty acid oxidation, and membrane potential), mitochondrial biogenesis, and mitochondrial integrity (Dinkova-Kostova and Abramov, 2015).

The association between fumarate and the Nrf2 pathway was based on indirect evidence from pathological models in which there was a deficiency in the FH enzyme which converts fumarate to malate in the Krebs cycle, with a subsequently increased concentration of fumarate. In these models (human FH-deficient cells and tissues, mouse embryonic fibroblasts, and Fh1 deficient renal cyst model (Adam et al., 2011; Ooi et al., 2011) an up-regulation of the Nrf2 pathway was observed, suggesting the potential succination of the Keap1 protein. The succination of Keap1 was later confirmed, and the specific modified sites which correspond to two critical cysteine residues were identified: Cys^155^ and Cys^288^. These modified sites disrupt the interaction of Keap1-Nrf2, resulting in stabilization and accumulation of Nrf2 in the nucleus, binding to AREs, and subsequent activation of target genes (Adam et al., 2011; Ooi et al., 2011). In this context, it is proposed that fumarate, by affecting the Nrf2 pathway, may contribute to neuronal protection, analogously to other cell types, by enabling them to tolerate different degrees of mitochondrial stress, thus promoting cell survival.

## Perspectives

Succination may induce the structural and functional alteration of cellular and extracellular proteins, and it offers a mechanism by which a Krebs cycle metabolite may lead to defects in the metabolism of neuronal and glial cells. Although it is not possible to offer comprehensive coverage of succinated proteome in the brain, advances in metabolomics and proteomics should yield further findings on the distribution at the subcellular, cell type, and regional levels of fumarate content and succinated proteome, as well as the cellular functions, affected. Systems biology will allow, in coming years, the development of an accurate view of the role of succination in defining and determining the selective vulnerability of all cell types that make up the human brain, as well as for an elucidation of the main factors in the brain aging process and, perhaps, neurodegeneration. Further studies are needed to consolidate these concepts.

## Conclusions

Succination is a non-enzymatic PTM of cysteine by the Krebs cycle metabolite fumarate, leading to the generation of 2SC, which is considered a biomarker of mitochondrial stress. There is a specificity of protein succination linked to structural and functional features. MS and antibody-based methods are the predominant approaches to detecting and quantifying protein succination, which occurs in brain tissue as in other cell systems. However, the concentration of 2SC in the human brain is region-specific. No changes have been detected in 2SC content in several brain regions throughout a healthy adult life span, suggesting sustained mitochondrial stress during aging which may support neuronal function and survival.

## Author Contributions

The manuscript was written by MJ, IP, NM-M, RC, VA, IF, and RP, and edited by RP.

## Conflict of Interest

The authors declare that the research was conducted in the absence of any commercial or financial relationships that could be construed as a potential conflict of interest.
